# Understanding the onset and remission of suicidal thoughts in Australian men: Findings from the Ten to Men study

**DOI:** 10.1177/00048674251333572

**Published:** 2025-04-16

**Authors:** Tilahun Haregu, Gregory Armstrong

**Affiliations:** 1 Centre for Mental Health and Community Wellbeing, Melbourne School of Population and Global Health, University of Melbourne, Melbourne, VIC, Australia; 2 Nossal Institute for Global Health, Melbourne School of Population and Global Health, University of Melbourne, Melbourne, VIC, Australia; 3 Baker Heart and Diabetes Institute, Melbourne, VIC, Australia

**Keywords:** Suicidal thoughts, first onset, remission, males

## Abstract

**Introduction:**

While Australian studies explore suicidal thoughts’ prevalence and correlates, little is known about their first onset in unaffected individuals or the predictors and rates of remission in the general population.

**Objective:**

The objective of the study is to determine the rates of first-onset suicidal thought and remission of suicidal thoughts among Australian males over a 9-year period and to identify predictors of these rates.

**Methods:**

This retrospective cohort study analysed 6035 participants from the Ten to Men study over four waves spanning 9 years. Outcomes included the first onset and remission of suicidal thoughts, with predictors encompassing sociodemographic, lifestyle, substance use, mental health and social factors. Modified Poisson regression with robust errors was used to identify predictors.

**Results:**

The rate of first-onset suicidal thoughts over the 9-year study period was 19.2% (95% confidence interval = [18.1%, 20.4%]). The remission rate for suicidal thoughts over the same period was 65.5% (95% confidence interval = [62.5%, 68.4%]). The onset of suicidal thoughts was linked to lower education, depressive symptoms, disability, financial stress, homosexual orientation and history of partner violence. Depressive symptoms and disability were also associated with reduced remission rates.

**Conclusion:**

One-fifth of males reported a first onset of suicidal thoughts, while nearly two-thirds with a history of such thoughts experienced remission over the 9-year period. Suicide prevention interventions need to prioritize males with a disability and mental health problems, as these factors both predict the first onset of suicidal ideation and reduce the rate of remission.

## Introduction

Findings from the 2020–2022 Australian Study of Mental Health and Wellbeing showed that one in six Australians (16.7% or around 3.3 million people) aged 16–85 had experienced serious thoughts about taking their own life at some point in their lives (Australian Bureau of Statistics (ABS), 2023). Evidence from the Longitudinal Study of Male Health in Australia reported that nearly 20% of Australian males have had suicidal thoughts in their lifetime, and about 10% of Australian males reported suicidal ideation in the past 4 weeks preceding the survey (Armstrong et al., 2020).

A longitudinal analysis of transitions in suicidal behaviour among Australian males has shown that between 7.6% and 10.5% of males aged 15–55 years with a history of suicidal thoughts and no prior history of suicide attempts will progress to a first suicide attempt within 2 years (Armstrong et al., 2023). A study on the Australian general population found that about 30% of those with suicidal thoughts had made suicide plans, and nearly a quarter had attempted suicide at some point. Most individuals who progressed to planning or attempting suicide did so within 2 years of their initial ideation. Factors such as alcohol and drug use disorders, major depression, obsessive-compulsive disorder, sexual minority status, and living in non-urban areas were linked to these transitions (Sunderland et al., 2023). In another study conducted among young women, 67.4% of respondents experienced remission of suicidal thoughts 17 months after the initial assessment, while 32.6% continued to have suicidal ideation at both assessments (Teismann et al., 2016).

The Leuven College Surveys and the Curtin Wellbeing Surveys, which are part of the World Mental Health International College Student Project, showed that non-suicidal self-injury is strongly associated with first-onset suicidal thought (Kiekens et al., 2018). A Canadian study found that three in five people with chronic pain who had once considered suicide were free of such thoughts in the past year. Those without recent ideation were more likely to be older, female, white, better educated, have a confidant, and use spirituality to cope while being less likely to face financial struggles or have a history of depression and anxiety (Fuller-Thomson and Kotchapaw, 2019).

While existing studies in Australia provide insights into the prevalence and correlates of suicidal thoughts in various population groups (Arya et al., 2024; Batterham et al., 2023; Kyron et al., 2021), little is known about the first onset of suicidal thoughts among individuals who initially do not experience them. In addition, there is limited evidence from representative, population-based longitudinal studies on the rates and predictors of the remission of suicidal thoughts at the general population level. Even at global level, most of the existing evidence on remission of suicidal behaviour is limited to clinical populations and specific population groups (Fuller-Thomson and Kotchapaw, 2019; Jollant et al., 2024; Teismann et al., 2016).

Evidence on the onset and remission of suicidal thoughts is crucial for informing health promotion and suicide prevention policies, especially for those at high risk of suicide-related mortality. By identifying when suicidal ideation typically begins and when it is most likely to subside, healthcare providers can better target their interventions to prevent suicide. Furthermore, understanding the specific predictors of both onset and remission – such as mental health conditions, socioeconomic factors, or social support systems – enables the development of targeted, evidence-based prevention strategies.

Using data from a large, population-based study of Australian males over a 9-year period, this study aims to address the existing evidence gap and enhance the understanding of the onset and remission of suicidal thoughts at the general population level. The objectives of this study were to determine the rates of first-onset suicidal thought among males without a history of suicidal thoughts and remission of suicidal thoughts among those who reported lifetime suicidal thoughts and to identify predictors of these rates among Australian males.

## Methods

### Data source

This study examined the onset and remission of suicidal thoughts among Australian males aged 10–55 over a 9-year period using data from four waves of the Ten to Men study. Details of the Ten to Men study have been reported elsewhere (Pirkis et al., 2016, 2017; Swami et al., 2022). In brief, Ten to Men is a longitudinal study that aims to survey the same group of males every few years. The first wave began in 2013 and collected health and lifestyle information from nearly 16,000 boys and men across the country through surveys and interviews. The second wave of data collection was conducted between 2015 and 2016. The third and fourth waves were collected in 2020 and 2022, respectively.

### Study population

The study population consisted of 6035 adult participants (18 years of age and above at wave 1) from the Ten to Men study who were followed up in all the four waves. This constitutes a follow-up period of 9 years for the cohort, spanning from 2013 to 2022.

### Study design

This study followed a retrospective cohort study design. To determine the rates of first suicidal thoughts, we followed males without a history of lifetime suicidal thoughts at wave 1 across the next three waves. To determine the remission rates of suicidal thoughts, we followed males with a history of lifetime suicidal thoughts at wave 1 across the subsequent waves.

### Study variables

#### Outcome variables

The *onset* of suicidal thoughts over the follow-up period was defined as the presence of newly developed suicidal thoughts at any of waves 2–4. This was assessed by a ‘Yes’ response to the lifetime suicidal thought question among those who reported ‘No’ to the same question at wave 1. This is an indirect approach to estimating the incidence of suicidal thoughts in the study population.

*The remission* of suicidal thoughts during the follow-up period was defined as the absence of suicidal thoughts in the past 12 months at all waves 2–4 among those who reported a lifetime history of suicidal thoughts at wave 1. This was assessed by a ‘No’ response to questions about suicidal thoughts in the past 12 months prior to the follow-up surveys, among those who reported ‘Yes’ to the lifetime suicidal thought question at wave 1.

#### Predictor variables

The great strength of the Ten to Men data set is that it contains a breadth of sociodemographic and health behaviour indicators, which are rarely available in data sets examining the risk of suicidality where the focus is more heavily on clinical factors. Our choice of variables was informed by theoretical models like the Interpersonal Theory of Suicide, which highlights factors like social isolation as being critical, as well as prior evidence on risk factors for suicide. Following an approach we used in a recent article (Armstrong et al., 2023), we have grouped the variables into the following categories: basic demographic factors, socioeconomic factors, health and substance use factors, and social and environmental factors. These factors, all measured at wave 1, include age in years, marital status, Aboriginal and Torres Strait Islander status, highest educational qualification, combined household income, employment status, region of residence, current smoking, depression symptoms (Patient Health Questionnaire−9 (PHQ-9)) (Kroenke et al., 2001), disability, alcohol use disorder (Alcohol Use Disorders Identification Test (AUDIT)) (Bohn et al., 1995), illicit substance use (including marijuana, cocaine, amphetamine and ecstasy), country of birth (categorized as Australia; AAME–Asia, Africa, Middle East; Anglo or European; and Others), main language spoken at home, having children, current smoking, night shift job, home ownership, financial stress, Socio-Economic Indexes for Areas (SEIFA), sexual orientation, history of partner violence, participation in religious services, participation in clubs, discrimination, social support using Medical Outcomes Study (Mościcki, 1995) scale (Sherbourne and Stewart, 1991) and personal well-being index.

### Data analysis

We used Stata 18.0 to analyse the data. The characteristics of the study participants were described using frequencies and proportions. The onset and remission rates of suicidal thoughts were also described by participant characteristics using frequencies and percentages. Chi-square tests were used to assess differences in the onset and remission rates of suicidal thoughts by participant characteristics. We employed modified Poisson regression with robust standard errors to identify predictors of the onset and remission of suicidal thought symptoms. We assessed multicollinearity using variance inflation factor (VIF). We reported the incidence rate ratios (IRRs) with the associated 95% confidence intervals and *p* values.

## Results

### Characteristics of study participants

A total of 6035 males who provided data in all four waves of the Ten to Men study were included in this analysis. Among these participants, more than half (51.2%) were aged 40−55 at wave 1, and nearly three-quarters (74.5%) were married. In addition, 9 of 10 were employed, and 3 of 5 lived in major cities at wave 1. Table 1 provides further details on the background characteristics of the study participants.

**Table 1.** Background characteristics and rates of new onset and remission of suicidal thoughts.

Background characteristics	Participants	New onset		Remission	
	*n* (%)^a^	*n* (%)^b^	*p* value	*n* (%)^b^	*p* value
Overall rates		889 (19.2)		687 (65.5)	
Demographic characteristics					
Age categories					
10−17 years	626 (10.4)	76 (28.3)	<0.001	34 (63.0)	0.962
18−29 years	950 (15.7)	172 (23.6)		137 (65.9)	
30−39 years	1367 (22.7)	204 (18.6)		174 (66.4)	
40−55 years	3092 (51.2)	437 (17.2)		342 (65.1)	
Marital status					
Never married	1074 (20)	182 (23.5)	<0.001	177 (61.7)	0.223
Divorced/separated/widowed	297 (5.5)	47 (23.5)		61 (66.3)	
Currently married	3996 (74.5)	576 (17.2)		412 (67.5)	
Aboriginal and/or Torres Strait islander					
No	5922 (98.7)	866 (19)	0.004	666 (65.6)	0.322
Yes	75 (1.3)	16 (36.4)		14 (56)	
Country of birth					
Australia	4866 (80.6)	758 (20.7)	<0.001	582 (64.9)	0.579
AAME-Asia, Africa, Middle East	475 (7.9)	41 (10.3)		34 (69.4)	
Anglo or European	426 (7.1)	44 (12.3)		43 (72.9)	
Others	268 (4.4)	46 (21.5)		28 (63.6)	
Language spoken at home					
English	5114 (95.1)	785 (19.1)	0.001	630 (65.4)	0.223
Other languages	262 (4.9)	24 (10.3)		20 (76.9)	
Sexual orientation					
Heterosexual	5317 (94.9)	814 (18.7)	0.003	626 (66.9)	0.013
Bisexual	77 (1.4)	14 (28.6)		17 (63)	
Homosexual	115 (2.1)	22 (34.4)		27 (52.9)	
Not sure	64 (1.1)	11 (25.6)		11 (61.1)	
Other	31 (0.6)	2 (8.3)		1 (14.3)	
Socioeconomic factors					
Highest educational qualification					
No completed qual	1040 (19.9)	190 (23.7)	<0.001	140 (61.4)	0.1
Certificate or diploma	2273 (43.4)	366 (20.2)		285 (64.3)	
Bachelor degree	1126 (21.5)	135 (14.2)		129 (74.1)	
Postgraduate	703 (13.4)	86 (14.4)		66 (65.3)	
others	95 (1.8)	13 (19.1)		17 (63)	
Employment status					
No	573 (10.6)	92 (25.3)	0.001	112 (55.7)	0.001
Yes	4815 (89.4)	717 (18)		541 (68.1)	
Combined household income					
Less than 40K	352 (7.1)	71 (30.2)	<0.001	67 (58.8)	0.14
40K to 79.999K	1133 (22.9)	166 (19.5)		191 (69.2)	
80K and above	3459 (70)	505 (17.3)		341 (66.3)	
Home ownership					
No	1348 (25.1)	220 (22.5)	<0.001	233 (64)	0.448
Yes	4013 (74.9)	588 (17.5)		409 (66.4)	
Financial stress					
No	4713 (78.1)	658 (17.5)	<0.001	428 (69.7)	0.001
Yes	1322 (21.9)	231 (26.5)		259 (59.5)	
SEIFA quartiles					
First	1187 (19.7)	186 (22)	0.007	176 (63.3)	0.78
Second	1572 (26)	238 (20)		192 (67.4)	
Third	1563 (25.9)	236 (19.7)		176 (65.2)	
Fourth	1713 (28.4)	229 (16.4)		143 (66.2)	
Health and substance use					
Alcohol use disorder					
No	3027 (64.3)	470 (18.6)	0.123	335 (69.6)	0.03
Yes	1677 (35.7)	266 (20.7)		238 (62.6)	
Depression (PHQ-9 ⩾ 10)					
No	4756 (89.4)	674 (16.7)	<0.001	501 (72.6)	<0.001
Yes	566 (10.6)	127 (46.5)		136 (47.4)	
Disability					
No	5320 (94.7)	800 (18.3)	<0.001	623 (68.2)	<0.001
Yes	297 (5.3)	62 (36.7)		58 (47.2)	
Marijuana use in the past year					
No	4486 (85.2)	668 (18)	0.001	488 (65.9)	0.832
Yes	777 (14.8)	132 (24.1)		146 (65.2)	
Ecstasy/cocaine use					
No	5038 (95.8)	755 (18.4)	0.016	594 (65.9)	0.107
Yes	219 (4.2)	42 (25.9)		31 (55.4)	
Current smoking					
No	4710 (87.7)	695 (17.8)	<0.001	517 (67)	0.122
Yes	663 (12.3)	115 (26.3)		135 (61.4)	
Amphetamine use					
No	5095 (96.8)	767 (18.4)	0.046	590 (65.6)	0.46
Yes	168 (3.2)	29 (25.9)		34 (60.7)	
Social and environmental factors					
Region of residence					
Major Cities	3600 (59.7)	505 (18.2)	0.026	407 (66.4)	0.074
Inner Regional	1376 (22.8)	204 (19.2)		148 (59.9)	
Outer Regional	1059 (17.5)	180 (22.5)		132 (69.8)	
Have children					
No	1580 (29.4)	271 (22.6)	<0.001	239 (64.9)	0.729
Yes	3799 (70.6)	537 (17)		412 (66)	
Job involves night shift					
No	3791 (79.3)	539 (17.1)	0.006	424 (69.2)	0.313
Yes	991 (20.7)	172 (21.3)		116 (65.2)	
Physically hurt by partner					
No	4450 (83)	637 (17.1)	<0.001	475 (67.2)	0.07
Yes	910 (17)	169 (27.3)		173 (61.1)	
Attend religious services					
No	3373 (56.4)	541 (21.3)	<0.001	433 (64.8)	0.546
Yes	2611 (43.6)	340 (16.5)		252 (66.7)	
Active member of club/association					
No	3069 (57.4)	466 (19.4)	0.126	408 (64.3)	0.274
Yes	2274 (42.6)	336 (17.5)		237 (67.7)	
Discrimination					
No	4433 (83.5)	639 (17.4)	<0.001	497 (67.8)	0.031
Yes	878 (16.5)	163 (25.9)		147 (60.2)	

*P* values are from chi-square tests.

a Column percentages.

b Row percentages.

### Onset and remission of suicidal thoughts

At wave 1, 4633 males (76.8%) reported no history of lifetime suicidal thoughts. Among them, the rate of first-onset suicidal thoughts over the 9-year follow-up period was 19.2% (95% confidence interval [CI] = [18.1%, 20.4%]), indicating that nearly one-fifth of males reported newly developed suicidal thoughts over the 9-year span. Among them, 12.0% had their first onset of suicidal thoughts between wave 1 and 2, and the rest 7.2% developed their first-onset suicidal thought between wave 2 and 4.

A total of 1049 (17.4%) of males included in this study reported lifetime suicidal thought at wave 1. The remission rate for suicidal thoughts among these males over the follow-up period was 65.5% (95% CI = [62.5%, 68.4%]), indicating that close to two-thirds of those with a history of suicidal thoughts at wave 1 did not report them again over the following decade. On the contrary, 34.5% reported suicidal thoughts in subsequent waves: 22.3% in one of the three waves, 8.1% in two of the three waves and 4.1% in all three waves.

Disaggregated analysis of the rates of first suicidal thoughts revealed significant variations across several participant characteristics at wave 1, including sociodemographic, health and social factors. The remission of suicidal thoughts varied based on employment status, alcohol use disorder, depressive symptoms and disability. Table 1 presents the onset and remission rates of suicidal thoughts by various participant characteristics.

### Predictors of onset and remission of suicidal thoughts

Higher educational status (having a bachelor’s degree) was associated with a 32% lower onset (IRR = 0.68; 95% CI = [0.53, 0.88]; *p* = 0.003), and depressive symptoms and disability were correlated with nearly a 74% (IRR = 1.74; 95% CI = [1.39, 2.19]; *p* < 0.001) and 44% (IRR = 1.44; 95% CI = [1.08, 1.92]; *p* = 0.013) higher incidence of suicidal thoughts, respectively. Financial stress and history of partner violence were associated with 22% (IRR = 1.22; 95% CI = [1.02, 1.46]; *p* = 0.030) and 39% (IRR = 1.39; 95% CI = [1.16, 1.67]; *p* < 0.001) higher onset of suicidal thoughts, respectively. Homosexual males were 2 times (IRR = 2.09; 95% CI = [1.36, 3.22]; *p* = 0.001) at higher risk of new onset suicidal thought as compared with heterosexual males.

Among participants who reported lifetime suicidal ideation at wave 1, depressive symptoms were linked to a 25% lower remission rate (IRR = 0.75; 95% CI = [0.61, 0.92]; *p* = 0.005) of suicidal thoughts. The presence of disability was associated with a 31% lower remission rate (IRR = 0.69; 95% CI = [0.48, 1.00]; *p* = 0.053) of suicidal thoughts. Details of predictors of onset and remission of suicidal thoughts are shown in Table 2.

**Table 2.** Predictors of onset and remission of suicidal thoughts.

Baseline predictors	Onset		Remission	
	IRR (95% CI)	*p* value	IRR (95% CI)	*p* value
Demographic Characteristics				
Age (years)	0.99 [0.98, 1]	0.052	1 [1, 1.01]	0.306
Marital status (Ref: Never married)				
Divorced/separated	1.33 [0.91, 1.94]	0.139	1.29 [1, 1.66]	0.052
currently married	1.23 [0.94, 1.62]	0.128	1.06 [0.88, 1.27]	0.552
Aboriginal_TSI (Ref: No)	1.59 [0.91, 2.79]	0.106	0.87 [0.54, 1.39]	0.552
Country of birth (Ref: Australia)				
AAME-Asia, Africa, Middle east	0.8 [0.53, 1.22]	0.299	0.94 [0.71, 1.24]	0.651
Anglo or European	0.73 [0.52, 1.03]	0.075	1.1 [0.9, 1.34]	0.366
Others	1.06 [0.75, 1.51]	0.744	0.81 [0.57, 1.15]	0.234
Language spoken at home (Ref: English)				
Other languages	0.78 [0.43, 1.41]	0.413	1.24 [0.9, 1.71]	0.188
Sexual orientation (Ref: Heterosexual)				
Bisexual	0.83 [0.46, 1.5]	0.536	1.09 [0.8, 1.49]	0.582
Homosexual	2.09 [1.36, 3.22]	0.001	0.81 [0.59, 1.12]	0.199
Not sure	1.98 [0.91, 4.32]	0.085	1.24 [0.73, 2.12]	0.425
Other	0 [0, 0]	0	0.55 [0.13, 2.3]	0.41
Socioeconomic Factors				
Highest educational qualification (Ref: High school or less)				
Certificate or diploma	0.89 [0.73, 1.08]	0.232	1.03 [0.86, 1.22]	0.755
Bachelor degree	0.68 [0.53, 0.88]	0.003	1.14 [0.95, 1.37]	0.161
Postgraduate	0.85 [0.65, 1.13]	0.265	1.02 [0.82, 1.27]	0.868
others	0.75 [0.37, 1.53]	0.43	1.09 [0.65, 1.83]	0.734
Combined household income (Ref: Less than 40K)				
40K to 79.999K	0.85 [0.62, 1.16]	0.296	1.05 [0.82, 1.35]	0.684
80K and above	0.89 [0.65, 1.22]	0.477	0.95 [0.74, 1.22]	0.687
Home ownership (Ref: No)	0.99 [0.82, 1.2]	0.949	0.95 [0.83, 1.08]	0.449
Financial stress (Ref: No)	1.22 [1.02, 1.46]	0.03	1.01 [0.88, 1.15]	0.934
SEIFA quartile (Ref: First quartile)				
Second	0.99 [0.8, 1.24]	0.963	0.95 [0.81, 1.11]	0.481
Third	1.01 [0.81, 1.26]	0.944	0.93 [0.79, 1.08]	0.342
Fourth	0.99 [0.78, 1.25]	0.906	0.97 [0.81, 1.16]	0.738
Health & Substance Use				
Alcohol use disorder (Ref: No)	1 [0.85, 1.18]	0.99	0.92 [0.81, 1.04]	0.191
Depressive symptoms (Ref: No)	1.74 [1.39, 2.19]	<0.001	0.75 [0.61, 0.92]	0.005
Disability (Ref: No)	1.44 [1.08, 1.92]	0.013	0.69 [0.48, 1]	0.053
Marijuana use (Ref: No)	0.89 [0.7, 1.12]	0.312	1.04 [0.88, 1.23]	0.627
Ecstasy/cocaine use (Ref: No)	1.35 [0.91, 2]	0.137	0.99 [0.73, 1.33]	0.932
Current smoking (Ref: No)	1.02 [0.8, 1.29]	0.892	0.99 [0.83, 1.18]	0.937
Amphetamine use (Ref: No)	0.77 [0.49, 1.23]	0.275	1.05 [0.78, 1.4]	0.752
PWI score	0.99 [0.98, 0.99]	<0.001	1.01 [1, 1.01]	0.028
Social & Environmental Factors				
Remoteness of residence (Ref: Major cities)				
Inner Regional	1.06 [0.88, 1.28]	0.563	0.94 [0.8, 1.09]	0.39
Outer Regional	1.16 [0.95, 1.41]	0.157	1.03 [0.88, 1.22]	0.684
Have Children (Ref: No)	0.87 [0.69, 1.09]	0.216	0.9 [0.76, 1.05]	0.176
Job involves night shift (Ref: No)	1.15 [0.96, 1.37]	0.125	0.91 [0.78, 1.06]	0.238
Hurt by partner (Ref: No)	1.39 [1.16, 1.67]	<0.001	0.9 [0.78, 1.04]	0.142
Attend religious services (Ref: No)	0.89 [0.75, 1.04]	0.139	0.99 [0.89, 1.12]	0.923
Active member of a club (Ref: No)	0.97 [0.83, 1.14]	0.748	0.99 [0.89, 1.11]	0.899
Discrimination (Ref: No)	1.09 [0.89, 1.34]	0.387	0.97 [0.84, 1.13]	0.696
MOS score^a^	1 [0.99, 1.00]	0.006	1 [1, 1]	0.07

a Estimates are rounded off.

## Discussion

Our study found that about one in five males without a history of suicidal thoughts reported experiencing them over the 9-year follow-up period. The main predictors of the incidence of first onset of suicidal thoughts were lower education, depressive symptoms, disability, financial stress, homosexual orientation and history of partner violence. Conversely, nearly two-thirds of males with a history of suicidal thoughts experienced remission during the same timeframe. Depressive symptoms and disability were significantly associated with reduced remission rates.

Evidence on the rates of new onset and remission of suicidal thought over a longer period is scarce. Most of the existing studies assessed these over a 1-year period, and our study extends the evidence in this area by having a 9-year follow-up period. Where suicidal thoughts were persistent for many men in our sample, it was nonetheless highly encouraging to note that for two-thirds of males with a history of suicidal thoughts there was no further experience of suicidal thoughts during the 9-year study period. Such findings are important in documenting that rate at which people with a history of suicidality can experience long periods of remission from suicidal thoughts, which can be used to offer hope to people in similar situations.

The identification of various risk factors such as homosexuality, depressive symptoms, disability, financial stress and history of partner violence as major contributors to first-onset suicidal thoughts among males in Australia is consistent with findings in the literature (Busby Grant et al., 2023; Peter et al., 2017; Marlow et al., 2021; Guan et al., 2022). Depression is one of the most significant predictors of suicidal ideation among Men. The presence of depressive symptoms can increase vulnerability to suicidal thoughts and behaviours (Kessler et al., 1999). Men with disabilities often face unique challenges, including social stigma, reduced access to healthcare, and barriers to employment, all of which can contribute to feelings of hopelessness and depression (Bishop et al., 2023). Studies have consistently shown that homosexual men face higher rates of mental health issues, including depression and anxiety, compared to their heterosexual counterparts. This can be attributed to experiences of discrimination, stigma, and social rejection and isolation, which can contribute to suicidal thoughts (Balakrishnan et al., 2022; Hill et al., 2021; Meyer, 2003).

Higher education levels often correlate with greater awareness of mental health issues, enabling individuals to recognize and seek help for suicidal thoughts or mental health challenges earlier (Kondirolli and Sunder, 2022). Educated men may have better access to resources and information about mental health. Men with higher educational attainment often have access to more extensive social networks and support systems. These connections can provide them with emotional support and practical help during difficult times, acting as protective factors against suicidal ideation (Rosoff et al., 2020).

Higher income can alleviate many stressors associated with financial insecurity, such as housing instability, food insecurity, and healthcare access (Thomson et al., 2022). Financial stress is a well-known risk factor for mental health issues, including depression and suicidal thoughts (Ryu and Fan, 2023). Men with higher incomes typically have better access to mental health services, including therapy and medication. This access can lead to early intervention and better outcomes for men experiencing mental health challenges. Higher income can provide men with the means to engage in activities that promote mental well-being, such as leisure activities, hobbies, and social interactions, which can act as buffers against stress and potential suicidal thoughts.

There are some limitations associated with this study. First, there was evidence of inconsistent reporting of lifetime suicidal thoughts, a situation where males who answered ‘yes’ to the lifetime suicidal thoughts question in wave 1 report ‘no’ in subsequent waves (17% in this study population), which may affect the robustness of our estimate of the first onset of suicidal thoughts. This is a well-documented phenomenon that is a challenge for longitudinal research on suicidal thoughts and behaviours (Klimes-Dougan et al., 2007). Second, the periods outside the 12 months prior to the surveys were not included in the analysis, as data on the history of suicidal thoughts beyond this 12-month period was not available. This may affect the generalizability of our findings on the remission of suicidal thoughts. Third, this study focused on predictors at wave 1. Changes in some predictors over time may affect new onset and/or remission of suicidal thoughts. Fourth, we included all males who reported lifetime suicidal thought at wave 1 regardless of the timing of the suicidal thought. The timing of suicidal thoughts – whether they occurred recently or a long time ago – may affect remission rates during the study period. Fifth, we included a variable for Aboriginal and Torres Strait Islander peoples as this sub-population is well-documented to be at higher risk of suicidality (Armstrong et al., 2017), yet the small size of this sub-group in our data meant this variable was under-powered. Finally, some variables in the multivariable model had high VIF values indicating risk of multicollinearity. Given their theoretical relevance, we retained these variables while acknowledging multicollinearity as a limitation.

In conclusion, one-fifth of males aged 18 and older reported the first onset of suicidal thoughts, while nearly two-thirds of those with a history of such thoughts experienced remission over a 9-year period. Depressive symptoms, disability, financial stress, sexual orientation and a history of partner violence were associated with a higher risk of developing suicidal thoughts, while higher educational status was associated with lower rates of new onset suicidal thoughts. In addition, depressive symptoms and disability were linked to lower rates of remission. Suicide prevention interventions should prioritize males with disabilities, financial stress, a homosexual sexual orientation, a history of partner violence, low educational qualifications and mental health conditions.

## References

[bibr1-00048674251333572] Australian Bureau of Statistics (ABS) (2023) National Study of Mental Health and Wellbeing (2020-2022). Canberra, ACT, Australia: Australian Bureau of Statistics.

[bibr2-00048674251333572] Armstrong G Haregu T Caine ED , et al. (2020) High prevalence of health and social risk behaviours among men experiencing suicidal thoughts and behaviour: The imperative to undertake holistic assessments. The Australian and New Zealand Journal of Psychiatry 54: 797–807. 32447979 10.1177/0004867420924098

[bibr3-00048674251333572] Armstrong G Haregu T Cho E , et al. (2023) Transition to a first suicide attempt among young and middle-aged males with a history of suicidal thoughts: A two-year cohort study. Psychiatry Research 328: 115445. 37666006 10.1016/j.psychres.2023.115445

[bibr4-00048674251333572] Armstrong G Pirkis J Arabena K , et al. (2017) Suicidal behaviour in Indigenous compared to non-Indigenous males in urban and regional Australia: Prevalence data suggest disparities increase across age groups. The Australian and New Zealand Journal of Psychiatry 51: 1240–1248. 28393536 10.1177/0004867417704059

[bibr5-00048674251333572] Arya V Burgess P Diminic S , et al. (2024) Suicidal ideation, suicide plans and suicide attempts among Australian adults: Findings from the 2020-2022 National Study of Mental Health and Wellbeing. Australian & New Zealand Journal of Psychiatry 10: 48674241256753. 10.1177/00048674241256753 PMC12102509 38859550

[bibr6-00048674251333572] Balakrishnan K Haregu T Hill AO , et al. (2022) Discrimination experienced by sexual minority males in Australia: Associations with suicidal ideation and depressive symptoms. Journal of Affective Disorders 305: 173–178. 35278485 10.1016/j.jad.2022.03.009

[bibr7-00048674251333572] Batterham PJ Gendi M Christensen H , et al. (2023) Understanding suicidal transitions in Australian adults: Protocol for the LifeTrack prospective longitudinal cohort study. BMC Psychiatry 23: 821. 37940886 10.1186/s12888-023-05335-1 PMC10634090

[bibr8-00048674251333572] Bishop GM Kavanagh AM Disney G , et al. (2023) Trends in mental health inequalities for people with disability, Australia 2003 to 2020. The Australian and New Zealand Journal of Psychiatry 57: 1570–1579. 37606227 10.1177/00048674231193881 PMC10666511

[bibr9-00048674251333572] Bohn MJ Babor TF Kranzler HR (1995) The Alcohol Use Disorders Identification Test (AUDIT): Validation of a screening instrument for use in medical settings. Journal of Studies on Alcohol 56: 423–432. 7674678 10.15288/jsa.1995.56.423

[bibr10-00048674251333572] Busby Grant J Batterham PJ McCallum SM , et al. (2023) Specific anxiety and depression symptoms are risk factors for the onset of suicidal ideation and suicide attempts in youth. Journal of Affective Disorders 327: 299–305. 36764362 10.1016/j.jad.2023.02.024

[bibr11-00048674251333572] Fuller-Thomson E Kotchapaw LD (2019) Remission from suicidal ideation among those in chronic pain: What factors are associated with resilience? The Journal of Pain 20: 1048–1056. 30979638 10.1016/j.jpain.2019.02.096

[bibr12-00048674251333572] Guan N Guariglia A Moore P , et al. (2022) Financial stress and depression in adults: A systematic review. PLoS ONE 17: e0264041. 10.1371/journal.pone.0264041 PMC8863240 35192652

[bibr13-00048674251333572] Hill AO Distefano A Gilmour S , et al. (2021) Social correlates of recent suicidal ideation among men who have sex with men in greater Tokyo. Sexuality Research and Social Policy 18: 467–478.

[bibr14-00048674251333572] Jollant F Demattei C Fabbro P , et al. (2024) Clinical predictive factors and trajectories of suicidal remission over 6 weeks following intravenous ketamine for suicidal ideation. Journal of Affective Disorders 347: 1–7. 37981038 10.1016/j.jad.2023.11.043

[bibr15-00048674251333572] Kessler RC Borges G Walters EE (1999) Prevalence of and risk factors for lifetime suicide attempts in the National Comorbidity Survey. Archives of General Psychiatry 56: 617–626. 10401507 10.1001/archpsyc.56.7.617

[bibr16-00048674251333572] Kiekens G Hasking P Boyes M , et al. (2018) The associations between non-suicidal self-injury and first onset suicidal thoughts and behaviors. Journal of Affective Disorders 239: 171–179. 30014957 10.1016/j.jad.2018.06.033

[bibr17-00048674251333572] Klimes-Dougan B Safer MA Ronsaville D , et al. (2007) The value of forgetting suicidal thoughts and behavior. Suicide and Life-threatening Behavior 37: 431–438. 17896883 10.1521/suli.2007.37.4.431

[bibr18-00048674251333572] Kondirolli F Sunder N (2022) Mental health effects of education. Health Economics 31: 22–39. 35797349 10.1002/hec.4565 PMC9796491

[bibr19-00048674251333572] Kroenke K Spitzer RL Williams JB (2001) The PHQ-9: Validity of a brief depression severity measure. Journal of General Internal Medicine 16: 606–613. 11556941 10.1046/j.1525-1497.2001.016009606.x PMC1495268

[bibr20-00048674251333572] Kyron MJ Rikkers W Page AC , et al. (2021) Prevalence and predictors of suicidal thoughts and behaviours among Australian police and emergency services employees. The Australian and New Zealand Journal of Psychiatry 55: 180–195. 32615800 10.1177/0004867420937774

[bibr21-00048674251333572] Marlow NM Xie Z Tanner R , et al. (2021) Association between disability and suicide-related outcomes among U.S. adults. American Journal of Preventive Medicine 61: 852–862. 34465506 10.1016/j.amepre.2021.05.035

[bibr22-00048674251333572] Meyer IH (2003) Prejudice, social stress, and mental health in lesbian, gay, and bisexual populations: Conceptual issues and research evidence. Psychological Bulletin 129: 674–697. 12956539 10.1037/0033-2909.129.5.674 PMC2072932

[bibr23-00048674251333572] Mościcki EK (1995) Epidemiology of suicidal behavior. Suicide and Life-Threatening Behavior 25: 22–35. 7631373

[bibr24-00048674251333572] Peter T Edkins T Watson R , et al. (2017) Trends in suicidality among sexual minority and heterosexual students in a Canadian population-based cohort study. Psychology of Sexual Orientation and Gender Diversity 4: 115–123. 29326961 10.1037/sgd0000211 PMC5758336

[bibr25-00048674251333572] Pirkis J Currier D Carlin J , et al. (2017) Cohort profile: Ten to men (the Australian Longitudinal Study on Male Health). International Journal of Epidemiology 46: 793–794i. 10.1093/ije/dyw055 27686951

[bibr26-00048674251333572] Pirkis J Macdonald J English DR (2016) Introducing ten to men, the Australian longitudinal study on male health. BMC Public Health 16: 1044. 28185546 10.1186/s12889-016-3697-2 PMC5103238

[bibr27-00048674251333572] Rosoff DB Kaminsky ZA Mcintosh AM , et al. (2020) Educational attainment reduces the risk of suicide attempt among individuals with and without psychiatric disorders independent of cognition: A bidirectional and multivariable Mendelian randomization study with more than 815,000 participants. Translational Psychiatry 10: 388. 33168806 10.1038/s41398-020-01047-2 PMC7653915

[bibr28-00048674251333572] Ryu S Fan L (2023) The relationship between financial worries and psychological distress among U.S. adults. Journal of Family and Economic Issues 44: 16–33. 35125855 10.1007/s10834-022-09820-9 PMC8806009

[bibr29-00048674251333572] Sherbourne CD Stewart AL (1991) The MOS social support survey. Social Science & Medicine 32: 705–714. 2035047 10.1016/0277-9536(91)90150-b

[bibr30-00048674251333572] Sunderland M Batterham PJ Calear AL , et al. (2023) Factors associated with the time to transition from suicidal ideation to suicide plans and attempts in the Australian general population. Psychological Medicine 53: 258–266. 33926588 10.1017/S0033291721001501

[bibr31-00048674251333572] Swami N Prattley J Bandara D , et al. (2022) Ten to Men: The Australian longitudinal study on male health: Waves 1–3. Australian Economic Review 55: 155–165.

[bibr32-00048674251333572] Teismann T Forkmann T Glaesmer H , et al. (2016) Remission of suicidal thoughts: Findings from a longitudinal epidemiological study. Journal of Affective Disorders 190: 723–725. 26600414 10.1016/j.jad.2015.09.066

[bibr33-00048674251333572] Thomson RM Igelström E Purba AK , et al. (2022) How do income changes impact on mental health and wellbeing for working-age adults? A systematic review and meta-analysis. The Lancet 7: e515–e528. 10.1016/S2468-2667(22)00058-5 PMC7614874 35660213

